# Science of Ventilation

**Published:** 1892-09

**Authors:** 


### SCIENCE OF VENTILATION.

The healthy atmosphere in a room is one in which the air is changed to the extent of 3,000 cubic feet per hour per adult inmate. The air admitted need not be cold; warmed air, so long as it is fresh, is of course preferable to cold air in winter, but in some way the air must be brought in if we are to continue in health. There are various ways of doing this. One is by admitting cold air so that it is directed upward toward the ceiling, where the air of a room is at the highest temperature; the cold stream is then heated in its passage as it falls to the lower level for breathing. But in large rooms, to utilize at its best this current, there should be in the skirting outlets communicating with a heated upcast flue, which will draw away the heavy air near the floor. In cases where there is heating by hot water coils, the cold air may be brought in at or near the floor level and passed through the hot water coils—the outlet for vitiated air being in or near the ceiling —to a heated upcast flue.

The great desideratum in the admission of fresh air is to cut it up into very fine streams, something in the way water is cut up in passing through the fine nose of a watering can. It has been found that air admitted through a tube or orifice of equal sectional area throughout enters as a cold draft, but if the inlet be through a series of small truncated cones, the smaller section outward, the larger inward, with a wire gauze on the inside, the current is so cut up and diffused that the draft is not felt. By analogy a mass of water entering through a narrow canal drives all before it and cuts a channel for itself, but the same quantity passing over a large surface of ground gently irrigates it. Another important point is not to let the passage of the air be at too great a velocity. The gentler the flow the better.

